# A Few Points in the Differential Diagnosis of the Skin

**Published:** 1917-01

**Authors:** Cosby Swanson

**Affiliations:** 929 Candler Bldg


### Journal-Record of Medicine

Successor to Atlanta Medical and Surgical Journal, Established 1855
and Southern Medical Record, Established 1870

**OWNED BY THE ATLANTA MEDICAL JOURNAL COMPANY**

PUBLISHED MONTHLY

*Official Organ Fulton County Medical Society, State Examing Board, Atlanta, Birmingham and Atlantic Railroad Surgeon's Association, Chattahoochee Valley Medical and Surgical Association, Etc.*

A. W. STIRLING, M. D., C. M., D. P. H.; J. S. HURT, B. Ph., M.D.
GEO. M. NILES, M. D., W. J. LOVE, M. D., (Ala.); Associate Editors

E. W. ALLEN, Business Manager

**COLLABORATORS**

W. F. WESTMORELAND, M. D., General Surgery
F. W. McRAE, M. D., Abdominal Surgery
ALLEN H. BUNCE, Medical Societies
E. B. BLOCK, M. D., Diseases of the Nervous System
MICHAEL HOKE, M. D., Orthopedic Surgery
CYRUS W. STRICKLER, M. D., Legal Medicine and Medical Legislation
E. C. DAVIS, A. B., M. D., Obstetrics
E. G. JONES, A. B., M. D., Gynecology
ALLEN H. BUNCE, M. D., Pathology and Bacteriology
R. T. DORSEY, Jr., B. S., M. D., Medicine
L. M. GAINES, A. B., M. D., Internal Medicine
GEO. C. MIZELL, M. D., Diseases of the Stomach and Intestines
L. B. CLARKE, M. D., Pediatrics
EDGAR PAULLIN, M. D., Opsonic Medicine
THEODORE TOEPEL, M. D., Mechano Therapy
E. G. BALLENGER, M. D., Diseases of the Genito-Urinary Organs

VOL. LXIII. ATLANTA, GA., JANUARY, 1917. NO. 10

### A FEW POINTS IN THE DIFFERENTIAL DIAGNOSIS OF THE SKIN.

### Manifestations of Syphilis From Other Dermatoses.

By Cosby Swanson, M. D.

The skin manifestations of syphilis at times present points of resemblance to almost every other dermatosis. This resemblance is so marked in certain skin diseases as to be very confusing and it is these conditions I desire to discuss.

The points of resemblance may be so great as to render it necessary to make a careful examination of the individual lesions, keep the patient under observation for several days and make laboratory investigations; such as blood tests and examination for the spirocheta pallida before a positive diagnosis can be made.

The eruption of syphilis may be either of a macular, papular, pustular, vesicular, bullous or mixed type. In rare cases it is polymorphous, but as a rule it is more uniform. When there are lesions of a variety other than those which make up the greater part of the eruption they are usually very few and scattered. The macular eruption of syphilis is usually the earliest skin manifestations of the disease to appear. In some cases the eruption is very scanty, especially early in the disease, later it becoms very abundant. In the early stages of the eruption the lesions may be a bright red color. After several days it is usually spoken of as being a dingy, muddy, dull-red or copper color. The lesions are oval, round or irregular in shape, varying in diameter from 1-4 inch to one inch.

The skin diseases that often have many striking points of resemblance to the eruption of syphilis are pityriasis rosea, tinea, versicolor, ringworm, drug eruptions, measles, rothelin, variola, keratosis pilaris, pityriasis rubra pilaris, lichen planus, papular eczema, psoriasis, acne vulgaris, leprosy, lupus vulgaris, carcinoma cutis and sporotrichosis.

The macular erupton of syphilis has to be differentiated from pityriasis rosea, perhaps more frequently than from any other skin diseases. The lesions of pityriasis rosea are usually irregular, circinate macules varying in size. The typical lesion has a reddish border with a red or yellowish branny formation of scales in the center, giving to the lesion a cigarette like crumpling. The majority of the lesions of pityriasis rosea have a tendency to increase in diameter and clear up in the center which give the lesion a slight elevated edge.

Pityriasis rosea differs from syphilis in that it is usually limited to the trunk, neck, upper part of the arms and thighs, rarely ever occurs on the forearms, hands, legs, feet, and never on the palms and soles.

The macular eruption of syphilis is usually most pronounced on the sides of the trunk, unbilical region, neck, flexor aspect of the forearms. The macular eruption of syphilis usually show a few scattered papules which is very rare in pityriasis rosea.

The diagnosis of syphilis from pityriasis rosea is usually easily made when all the symptoms of syphilis are taken into consideration, such as, sore throat, mucous patches, moist papules, falling out of the hair; in most cases the history or presence of a primary lesion, a positive Wassermann reaction, and the presence of the spirocheta pallida.

The diagnosis of tinea versicolor from syphilis is often confusing in that it has many of the characteristics of the macular eruption of pigmented syphilides. Tinea versicolor begins as minute rounded macules, located on the chest, back shoulders, abdomen and axilla. The lesions gradually increase in size and number until they usually involve a large area. The lesions are yellow or brownish-yellow in color and the surface is covered with furfuraceous scales. A positive diagnosis from pigmented syphilis can be made by the aid of the microscope, finding the microsporon furfur.

Chloasma is very often mistaken for pigmented syphiloderm. The patches of chloasma are usually localized, being round or irregular in shape, covering a large area with ill defined margins. In chloasma the parts most frequently involved is the face, forehead and malor prominences. In syphilis the eruption usually occurs on the neck, shoulders and only in rare cases on other parts of the body. The lesions of syphilis consist of pin-head-to-pea sized or larger, rounded or oval, ill defined pigment-free patches with hyperpigmented areoloa. The majority of the cases occur in young women, while chloasma occurs most frequently after middle life. Chloasma can usually be diagnosed from syphilis by the shape and size of the lesions, together with the absence of any other symptoms of syphilis.

The eruption of rubeola and rubella often resembles the early macular papular syphiloderm. The majority of cases of rubeola can be differentiated from syphilis by the acute onset, the catarrhal symptoms, elevation of temperature, crescentic and blotchy character of the lesions, distribution of the eruption, Koplik's patches, the absence of the primary lesion, and negative Wassermann reaction· Rubella differs from syphilis in that the onset is more abrupt and of a shorter duration. The lesions are smaller, more confluent, redder in color, with no tendency to pigmentation on disappearing.

Pustular syphiloderm often has to be differentiated from variola. The eruption of variola differs from pustular syphiloderm in that the lesions are deeper seated, firmer with thicker walls, a larger number are pustulated and umbilicated. The eruption of variola develops more rapidly, usually in the course of a few hours, accompanied by more catarrhal symptoms and fever.

The eruption of variola involves primarily the forehead, the flexor surface of wrists. In syphilis the lesions usually appear irregularly, requiring one, two or more weeks, while in variola all the eruption appears about the same time.

The diagnosis of pustular syphiloderm from variola can usually be made by the history, character of lesions, distribution of the eruption, serum reaction, etc.

The drug eruptions that often have to be differentiated from the pustular lesions of syphilis are those due to the action of the iodides and bromides. The eruptions caused from the action of the iodides and bromides generally appear suddenly and are usually mild in character. In some cases they are verv severe from the beginning, due to an idiosyncrasy of the drugs.

The drug eruptions differ from syphilitic eruptions in that they are redder, more discrete and more scattered, especially in the beginning. The drug lesions generally appear while taking the drugs and disappear when the drugs are discontinued. The drug eruptions usually occur first on the face and shoulders, while the eruption of syphilis occurs on any part of the body. The eruption of syphilis is usually more evenly distributed.

The late eruption caused from the action of iodides and bromides have a tendency to group formation and to become more confluent, forming a sluggish condylomatous like patches in which are scattered pustules. The diagnosis of syphilis from iodide and bromide eruptions can usually be made by the history, distribution of the lesions, serum reaction and the absence of other symptoms of syphilis.

Acne vulgaris often has to be differentiated from the pustular form of syphilis, especially when it occurs on the shoulders, chest, back and buttocks. Acne differs from syphilis in that it is usually limited to the face, chest, shoulders, back and buttocks, while syphilis occurs on other parts of the body.

In acne comedones, papules, pustules and often nodules are found in all stages of development. In syphilis the lesions are more uniform in size and type. Comedones are never found unless acne is a complication. Acne is more sluggish in character, and does not as a rule yield to antisyphilitic treatment. The differential diagnosis of acne from syphilis can usually be made by the age of the patient, history, distribution of the eruption, nature of the lesions, serum reaction, treatment and absence of any other symptom of syphilis.

The differential diagnosis of acne rosacea from syphilis is often confusing, especially cases in which a few scattered nodules are present. Acne rosacea differs from syphilis in that there is more congestion, the sebaceous glands are more active and enlarged. In rosacea, seborrhoeic dermatitis is a frequent complication and this seldom occurs in syphilis.

The hypertrophic nodules found in acne rosacea differs from nodular syphiloderm in that it does not have a tendency to ulcerate, and on healing leaves less scarring and atrophic changes. Rosacea can usually be differentiated from syphilis by the history, color, dilated capillaries; limitation of the eruption to the flush areas and forehead, persistent nature of the disease, negative Wassermann reaction and negative therapeutic test.

The lesions of lichen planus often resembles the lesions of papular syphilis. The lesions of lichen planus differ from syphilis in that they are usually more irregular in shape with flat tops and a glistening shiny violaceous color, while the lesions of syphilis are usually pointed with a dull copper color. In lichen planus many of the lesions are unbilicated with a small scale in the center. Lichen planus has a tendency to patch-formation, while in syphilis the distribution is more general. In lichen planus pustular lesions never occur and occur frequently in syphilis. The diagnosis of lichen planus from papular syphilis can be made by the history, nature, character and distribution of the eruption, serum reaction.

Keratosis pilaris and pityriasis rubra pilaris often resembles the miliary papular eruption of syphilis. In keratosis pilaris the eruption differs from syphilis in that it is less inflammatory, the itching is greater, the lymph-modes are not involved and the mucous membranes are not affected. In keratosis pilaris, the eruption usually occurs on the scalp, forearms, lateral aspect of the thighs, while in syphilis the distribution of the eruption usually is general. The lesions of keratosis pilaris are more pointed, usually smaller in size with a whitish or grayish center and usually encircles a hair shaft.

Pityriasis rubra pilaris differs from syphilis in that it begins insidiously on the scalp, palms and soles and after some weeks or months, becomes general, while the eruption of syphilis generally begins abruptly on the body, becoming general in a few days. Pityriasis rubra pilaris and kerotosis pilaris can usually be differentiated from syphilis by the history, character of the lesions, nature and distribution of the eruption, therapeutic test and absence of other symptoms of syphilis.

The diagnosis of the nodular form of syphilis from other nodular dematoses at times becomes very difficult. Nodular syphilides usually occurs late in the course of the disease, seldom before the end of the first year. They are as a rule limited in number with a tendency to grouping, forming circinate and serpiginous configurations. The lesions of syphilis in the beginning are bright red in color, smooth, round, circumscribed elevations, with a firm consistency· Later they become dull red in color, less firm, and are covered with thin exfoliating scales.

Lupus vulgaris differs from nodular syphiloderm in that it usually begins early in life while syphilis begins in middle or late life. Lupus usually occurs on the face, rarely ever on any other part of the body, while syphilis usually occurs on the face and body at the same time. The lesions of lupus are more of a yellowish-red or brownish-red color, while that of syphilis are dark red, more of a copper color. Lupus is very slow in its course while syphilis often is rapid.

The ulceration of lupus is usually more superficial than that of syphilis. The discharge in lupus when present is scanty, while in syphilis it is profuse. In lupus, apply-jelly colored tubercules are usually found and are never found in syphilis. In cases of doubt it is much safer to make a diagnosis of syphilis rather than lupus unless there are other conclusive reasons for making a diagnosis of lupus. The diagnosis of syphilis from lupus can usually be made by the history, character of the lesions, serum reaction, therapeutic test and a careful examination of the body which will often disclose other valuable symptoms when syphilis is present.

Nodular syphilis differs from carcinoma cutis in that it usually begins in multiple lesions, while carcinoma usually begins in single lesions. Syphilitic nodules, are more superficial than carcinoma, ulcerate quicker and are not as a rule round as are the lesions of carcinoma. The lesions of syphilis at times become segmented, irregular or circinate in shape which rarely ever occurs in carcinoma. Syphilis usually occurs in middle life, while carcinoma occurs more often in advanced life. The lesions of syphilis does not assume the distended bright red color or brownish, bluish or purplish tinge as carcinoma. When ulceration occurs in carcinoma, the patient gradually drifts into a cachectic condition and dies. The differential diagnosis of syphilis from carcinoma can usually be made very easily by the history and the pathological findings.

The diagnosis of psoriasis from syphilis is often difficult, especially the squamous form. The early manifestations of psoriasis differs from syphilis, in that the lesions are brighter red in color, occur more frequently on the back, buttocks and extensor surfaces, while the lesions of syphilis occurs more frequently on the face, elbows, knees and flexor surfaces. In psoriasis the lesions gradually increase in size and number, and never ulcerate, while in syphilis they seldom increase in size and often ulcerate. In syphilis macerated lesions are usually found in the genital, gluteal, axillary regions, and are never found in psoriasis. The scales of psoriasis have a pearly appearance, while in syphilis they are rough and of a dull yellow color. The lesions of syphilis usually are few in number, occur asymmetrically while in psoriasis they occur bilaterally. In syphilis the lesions heal with more or less scarring while in psoriasis scarring never occurs. Psoriasis can usually be differentiated from syphilis by the history, color of the lesions, character of the scales, serum reaction, and the absence of other symptoms of syphilis.

The diagnosis of syphilis from the nodular form of leprosy are at times difficult. Syphilis differs from leprosy in that the lesions usually pursue a comparatively rapid course, while the lesions of leprosy develop very slowly. In syphilis the lesions are a dull red or copper color, while in leprosy they are more of a yellowish or bluish color. In leprosy, pigmented patches of anesthenia often occur along with the nodules and never occurs in syphilis.

Leprosy is very seldom seen in this section, while syphilis is seen often. The blood of both diseases usually gives a positive reaction to the Wassermann test. In both diseases the mucous membrane of the mouth is involved with equal frequency. The diagnosis of syphilis from leprosy can be made by the laboratory findings; in suspected cases of syphilis by finding the spirocheta pallida, and in suspected cases of leprosy the lepra bacillus.

The gummatous lesions of syphilis often resembles fatty, fibrous and malignant tumors, sebaceous cysts, enlarged lymph-nodes and carbuncles.

Gummata differs from other similar lesions in that the ulceration is deeper seated with sharper and more punched out edges and less infiltration.

Epithelioma differs from syphilis in that there are usually several small pearl-like papules or nodules on the side of the lesion, usually giving them a simitranslucent appearance. The diagnosis of gumma from other similar growths can usually be made by the history, color, nature of the growth, serum reaction, supplemented by the microscopical examination of the tissue.

The lesions of erythema induratum differs from nodular syphiloderm in that they develop slower, always occur on the legs bilaterally, while the lesions of syphilis, usually are found on other parts of the body; often occur in groups and usually unilaterally. Erythema induratum does not as a rule yield to antisyphilitic treatment.

The diagnosis of sporotrichosis from syphilitic gummata are often difficult. In sporotrichosis the lesions usually ulcerate early, while in syphilis, ulceration rarely ever occurs, when it does occur it is late in the disease. In sporotrichosis, a chain of nodules appear following the course of the lymphatics and this seldom ever occurs in syphilis. When ulceration occurs in sprorotrichosis there is a profuse purulent discharge of a yellowish or brownish color. In sporotrichosis the lesions are more persistent with a greater tendency to multiply. Sporotrichosis and syphilis are alike in that both heal with scar formation, and yield to the action of the iodides. Sporotrichosis can usually be differentiated from syphilis by the history, distributión of the lesions and laboratory findings. In sporotrichosis the parasite grows readily on ordinary culture media. The culture media can be inoculated from the lesions or from the blood of individuals suffering from the disease. In cultures, the organisms consist of long slender mycelia and minute, rounded or oval spores. --

929 Candler Bldg

